# Diagnosis, management, and recovery from COVID‐19: A case report from bangladesh

**DOI:** 10.1002/ccr3.3895

**Published:** 2021-02-11

**Authors:** Lutfa Akther, Shaila Sharmin Shahnewaz, Dilruba Ferdous, Md. Moyen Uddin Pramanik

**Affiliations:** ^1^ RHSTEP Dhaka Medical College and Hospital Dhaka Bangladesh; ^2^ Physical Medicine and Rehabilitation Narayanganj Diabetic Hospital Narayanganj Bangladesh; ^3^ Dhaka Medical College and Hospital Dhaka Bangladesh; ^4^ Institute of Biological Science Rajshahi University Rajshahi Bangladesh; ^5^ Biochemistry & Molecular Biology Rajshahi University Rajshahi Bangladesh; ^6^ Primeasia University Dhaka Bangladesh; ^7^ Independent University Dhaka Bangladesh; ^8^ Institute for Research in Molecular Medicine Universiti Sains Malaysia Malaysia Malaysia

## Abstract

There is no known medicine for COVID‐19, and thus, the clinical management is symptomatic. More than 80% people experience mild symptoms who can be treated at home for fever, sore throat, and cough. About 15% patients develop severe illness require O2.

## Graphical abstract

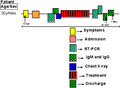

## 1 INTRODUCTION

A 30‐year‐old man with no travel history to COVID‐19–prone areas had the characteristic signs and symptoms of COVID‐19. Chest X‐ray and RT‐PCR confirmed his COVID‐19 diagnosis. This study recommends the early identification and management of COVID‐19.

Coronavirus disease (COVID‐19), caused by severe acute respiratory syndrome coronavirus 2 (SARS‐CoV‐2), was first identified in Wuhan, China, in December 2019.^1^ The World Health Organization (WHO) declared a public health emergency of international concern regarding this global outbreak on 30 January 2020. Globally, there have been 5 796 257 confirmed cases and 362 483 deaths from COVID‐19 reported to the WHO as of 9:37 AM CEST on 30 May 2020.^2^ In Bangladesh, there have been approximately 52 000 confirmed cases of COVID‐19 with about 700 deaths as of 4 June 2020. The SARS‐CoV‐2 virus is primarily transmitted between humans through respiratory droplets and contact routes. Analysis of 75 465 COVID‐19 cases in China failed to detect airborne transmission.^3^, ^4^, ^5^ Early disease symptoms offer an opportunity for early detection.^6^, ^7^ Fast identification and accurate treatment have been critical to prevent the spread of infection.^8^, ^9^ At present, reverse transcription polymerase chain reaction (RT‐PCR) and real‐time RT‐PCR (qRT‐PCR) are used to detect SARS‐CoV‐2. There are three genes expressed by SARS‐CoV‐2, including the RNA‐dependent RNA polymerase (RdRp)/helicase (Hel), spike (S), and nucleocapsid (N) genes^10^, ^11^ In Bangladesh, qRT‐PCR is used to detect early COVID‐19. After a week, point‐of‐care test kits for antigen and antibodies are used to measure the protective level of plasma IgM and IgG antibodies. In this study, we reported a case of COVID‐19 in a man with no travel history to COVID‐19–prone areas, yet he had the characteristic signs and symptoms of COVID‐19. Chest X‐ray and qRT‐PCR confirmed his COVID‐19 diagnosis.

## 2 CASE HISTORY AND EXAMINATION

**FIGURE 1:**
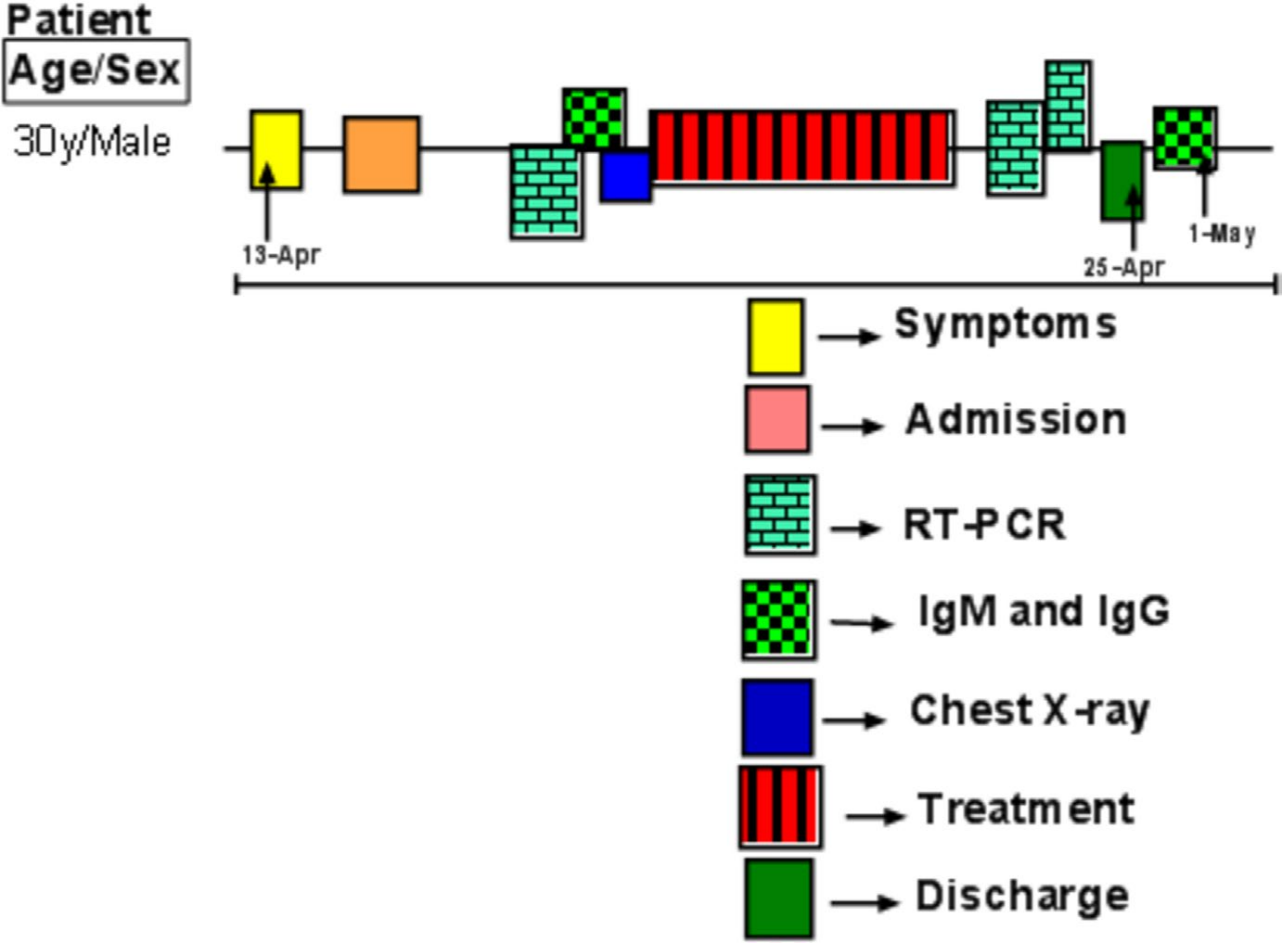
Snapshot of COVID‐19 in a 30‐year‐old man

On 13 April 2020 at 11:30 AM, a 30‐year‐old man presented to a tertiary hospital in Dhaka, Bangladesh, with the signs and symptoms of COVID‐19. He had dry cough, muscle pain, fever, headache, and shortness of breath. He was immediately admitted and recorded the vital signs of COVID‐19, which are shown in Table 1. He had no history of direct contact with any COVID‐19–positive individuals. On 14 April at 10:15 PM, his COVID‐19 diagnosis was confirmed using an RT‐PCR nasal swab test (Table 2). At 12:45 AM, he was moved to an isolated cabin and received supportive treatment. On 15 April at 9:45 PM, we received his complete blood count results, which did not identify leukocytopenia. Chest X‐ray, on the other hand, showed ground‐glass opacities in the center and lower right portion of his lung (Table 2). This finding strongly indicated COVID‐19. On 16 April at 10:30 AM, new nasal and throat swabs were tested for SARS‐CoV‐2 by RT‐PCR, and the findings were positive. At 11:35 PM, he was treated with antibiotics (azithromycin, 500 mg), O_2_ inhalation, intravenous fluids, acetaminophen (500 mg), montelukast (10 mg), and fexofenadine (120 mg). After 3 days of treatment (19 April 2020), his fever and breathing improved, and his condition was stable for 72 hours. On 23 and 24 April, he was retested for SARS‐CoV‐2 RNA with two independent throat swabs; both were negative (Table 2). On 25 April at 11:25 AM, he was discharged and recommended to follow the requirements to home quarantine for the next 14 days. During his 1 May 2020 follow‐up, it was recommended that he monitors his blood IgM and IgG against SARS‐CoV‐2. Antibodies (IgM and IgG) were measured using a point‐of‐care test, which detected a protective level of IgG (Table 2). The snapshot of COVID‐19 is shown in Figure 1.

**TABLE 1:** Vital signs of COVID‐19 recorded on admission

Vital signs	Response
Body temperature (^o^F)	102.7
Blood pressure (mm Hg)	110/75
Heart rate (beats/min)	93
Respiratory rate (breaths/min)	18
Oxygen saturation (%)	86
Sore throat	Mild
Rhinorrhea	N
Diarrhea	N
Cough	Moderate
Anosmia	N
Chest pain and tightness	N
Muscle pain	Mild
Chill	N

Abbreviations: N; none, mmHg; A millimeter of mercury.

**TABLE 2:** SARS‐CoV‐2 RNA detections by Reverse Transcriptase Polymerase Chain Reactions

Date	RT‐PCR		IgM/IgG	X‐ray
	Nasal swab	Throat swab	Blood	Chest
14 April 2020	Positive			Ground‐glass darkness in the center and lower right of the lung
15 April 2020				
16 April 2020	Positive	Positive		
23 April 2020		Negative		
24 April 2020		Negative		
1 May 2020			IgM−/IgG+	

Abbreviations: Ggd; ground‐glass darkness; IgG, immunoglobulin G; IgM, immunoglobulin M; RT‐PCR; reverse transcriptase polymerase chain reactions.

## 3 DISCUSSION

Severe COVID‐19 illness begins after approximately 7 days after symptom onset. The most common symptoms of COVID‐19 are fever, cough, tiredness, dyspnea, and diarrhea.^12^, ^13^ Severe COVID‐19 dyspnea triggers progressive respiratory failure followed by severe hypoxemia. In addition, lymphopenia is a frequent symptom of severe COVID‐19.^14^, ^15^ COVID‐19 is diagnosed and monitored using qRT‐PCR, partial or complete viral genome sequencing, and plasma antibody‐level measurements. Early SARS‐CoV‐2 is detected by collecting, processing, and analyzing respiratory samples, such as nasopharyngeal aspirates, combined (nasal and oral) swab samples, sputum, tracheal lavage fluid, or bronchoalveolar lavage fluid.^16^ RNA is isolated from respiratory samples and measured utilizing qRT‐PCR with SARS‐CoV‐2–specific primers.^17^ Within a week of COVID‐19–symptom onset, plasma IgM and IgG tests easily detect the infection. It showed that a patient had the protective amount of IgG within 14 days. Point‐of‐care tests can identify serum antibodies in 15 minutes and can determine the level of the infection.^18^ Computed tomography is useful for both diagnosis and follow‐up. Currently, there is no proven successful treatment for COVID‐19. Antiviral medications and respiratory treatment are the primary approaches used to cure the disease. In addition, while several therapies have been suggested, quarantine is the only method that seems to be successful.

## 4 CONCLUSION

The most critical aspect of treatment in this case study was close observation of the patient's respiratory condition to assess whether a ventilator was necessary. The patient was recommended to quarantine at home for 2 weeks (14 days) with proper food management. This 2‐week period provides adequate time to determine whether or not they have been infected and are able to spread the infection to other citizens.

## CONFLICT OF INTEREST

The authors declare that there are no conflicts of interest.

## AUTHOR CONTRIBUTIONS

Lutfa Akther, Shaila Sharmin Shahnewaz, and Dilruba Ferdous: were involved in data collection and reviewed the manuscript. Md. Moyen Uddin Pramanik: analyzed the data and wrote the manuscript.

## ETHICAL APPROVAL

This case study does not include any scientific trials involving human subjects.

## Data Availability

The data that support the findings of this study are available from the corresponding author upon reasonable request.
